# Qualitative study on the ability of neurological nurses to manage patients with indwelling gastrointestinal canal

**DOI:** 10.3389/fmed.2024.1403173

**Published:** 2024-12-03

**Authors:** Zezhou Wang, Yan Liu, Kui Fang, Xixi Hao, Zhihan Xue, Xue Dong, Haijun Wang

**Affiliations:** The First Affiliated Hospital of China Medical University, Shenyang, China

**Keywords:** enteral nutrition, neurology, nurse, gastrointestinal intubation, qualitative research

## Abstract

**Aim and objectives:**

This study aims to assess the proficiency of nurses in the neurology department in managing patients with indwelling gastrointestinal canal.

**Background:**

Many critically ill patients in neurology departments require the placement of nasoenteric tubes to provide enteral nutrition. However, in clinical practice, there exists considerable variability in nurses’ competencies regarding the management of patients with nasoenteric tubes. A thorough understanding of the challenges nurses face throughout the entire process of nasoenteric tube care and the development of targeted improvement measures are crucial for delivering enhanced patient care and facilitating patient recovery.

**Design:**

A qualitative interview study.

**Methods:**

From August to October 2023, 11 neurological nurses were recruited offline in three hospitals for semi-structured and in-depth interviews. The data analysis uses the KJ method to summarize and classify the themes from the semi-structured interview results, and then organize the themes into a coherent visual and logical path. The COREQ list is used.

**Results:**

The researchers conducted semi-structured interviews with 11 neurology nurses from three hospitals using a purposive sampling method, of which 64% of the participants were female and 36% were male; the average age was 38.27 ± 7.85 years; 27% from the Department of Neurology, 73% from the Department of Neurosurgery; junior professional titles accounted for 18%, intermediate professional titles accounted for 64%, senior professional titles accounted for 18%; 27% of the subjects were head nurses and 73% were nurses. The study identified 5 themes: (1) Preparation and evaluation before intubation; (2) Enhancement of intubation success rates; (3) Prevention of complications; (4) Management of complications and emergencies; (5) The acquisition of relevant knowledge and experience. In clinical practice, the bedside blind insertion technique is random and has not yet formed a unified standard, and the specific operation details and techniques need to be further improved.

**Conclusion:**

Disparities exist in the nursing capabilities of neurology nurses due to variations in their knowledge and experience regarding gastrointestinal canal management.

## 1 Background

Neurological patients often experience limitations in oral nutrition intake due to swallowing dysfunction, accompanied by gastrointestinal issues and altered consciousness. These factors not only hinder nutrient absorption but also contribute to metabolic dysfunction and malnutrition (1). Consequently, they impede the recovery of neurological functions (2). According to guidelines (3), early intervention using post-pyloric enteral nutrition preparations is recommended for patients unable to orally ingest, exhibiting feeding intolerance, or at risk of aspiration due to altered consciousness. Catheterization for post-pyloric access can mitigate feeding intolerance, prevent aspiration pneumonia resulting from gastroesophageal reflux, and decrease adverse feeding events (4, 5). The timely application of gastrointestinal canal (6) is deemed an effective method for postpyloric pathway catheterization (7). Nasointestinal tube feeding offers advantages such as non-invasiveness, simplicity of use, high safety, comfort, and the ability to minimize the risk of aspiration to a significant extent (8). This nutritional pathway, inserted into the duodenum or jejunum through the nasal cavity, pharynx, esophagus, and stomach, provides patients with essential nutrients, fluids, medications, etc., fulfilling the body’s requirements for water, electrolytes, and intestinal decompression. It is widely utilized in clinical settings (9).

Neurological nurses are required to communicate and coordinate with a multidisciplinary team (10), including physicians, dietitians, patients, and their families, when managing patients with indwelling nasoenteric tubes. However, nurses often lack the necessary knowledge and skills for the management of nasoenteric tubes, including tube placement (11), maintenance (12), and removal (13). This deficiency may increase the risk of complications such as tube placement failure, infection, and malnutrition (14). A previous study (15) has revealed discrepancies between nurses’ enteral nutrition practices and current best evidence. Moreover, research (16) has revealed that nurses face numerous challenges in caring for patients with nasoenteric tubes, such as difficulties with tube insertion and a lack of training related to enteral nutrition. In developing countries like China, there is limited understanding of nurses’ experience in selecting the appropriate type of nasoenteric tube, improving the success rate of insertion, and preventing and managing related complications (17). Ma et al. (18) found that many Chinese nurses have not received adequate training in nasoenteric tube management and may lack systematic education and practical experience in this area, even when encountered in clinical practice. To address these issues, it is essential to understand the challenges faced by neurological nurses in managing patients with indwelling nasoenteric tubes and to enhance the relevant education and training for nurses in nasoenteric tube management. This should include the implementation of clinical guidelines and the improvement of working environments and resource allocation. By taking these measures, the ability of neurological nurses to manage patients with indwelling nasoenteric tubes can be improved, thereby enhancing patient safety and satisfaction.

This study applies the KJ method (19) to a qualitative investigation of the proficiency of neurological department and neurosurgery nurses in managing patients with indwelling nasojejunal tubes. It delves deeply into issues related to nurses’ capabilities in caring for patients with indwelling nasojejunal tubes, aiming to provide a reference basis for further standardizing clinical practices.

## 2 Methods

### 2.1 Study participants

This study utilized purposive sampling for data collection. From August to October 2023, neuroscience nurses were recruited through offline methods at the First Affiliated Hospital of China Medical University, the Shengjing Hospital Affiliated with China Medical University, and the Liaoning Provincial Cancer Hospital. Sample selection adhered to the principle of maximum variation, with the sample size being determined based on data saturation.

Inclusion criteria: (1) Informed consent and willingness to participate in the study. (2) Proficient language expression and communication skills. (3) The nurses directly cared for the patients and managed the nasojejunal tube.

Exclusion criteria: Nurses in practice.

### 2.2 Formulation of interview outline

The research group consisted of seven members, including a professor who had studied the KJ method in Japan, an expert in enteral nutrition nursing research, and five nursing postgraduates.

On the basis of searching and analyzing the literature, to understand the current situation of the ability of neurology nurses to manage patients with indwelling nasointestinal tube, and to preliminarily determine the content of the interview outline. After pre-interview with two nurses, the outline was refined to form a formal interview guide. The finalized questions include:

① Which neurological patients do you believe require indwelling nasointestinal tubes? ② What preparations do you believe are necessary for both the patient and the nurse prior to tube insertion? ③ How can the success rate and efficiency of tube placement be enhanced? ④ How should care be provided during the period a nasoenteric tube is in place? ⑤ How can complications related to the nasoenteric tube be managed scientifically and effectively?

### 2.3 Data collection method

Two team members collected qualitative research data experienced in qualitative research through semi-structured interviews. Ethics Committee approval was obtained. Before the interviews, participants were briefed on the study’s purpose, content, and significance, informed about the voluntary nature and confidentiality of their participation, and provided informed consent. The entire interview process was meticulously recorded. Observations were conducted to note changes in the interviewees’ facial expressions, body language, intonation, and emotional variations. Any ambiguities or unclear content were immediately addressed through rephrasing or further questioning for clarification. Each interview was conducted within a timeframe of 20 to 30 min.

### 2.4 Data analysis method

**Figure 1:**
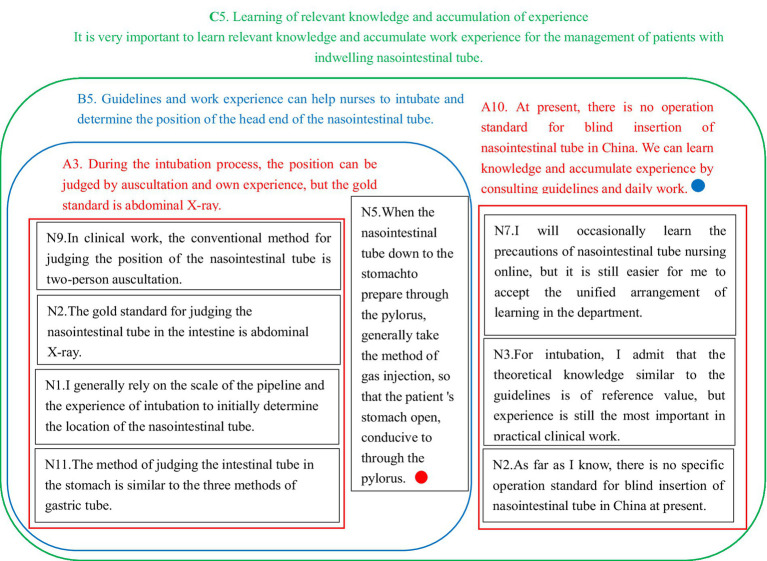
An example diagram of KJ analysis.

**Figure 2:**
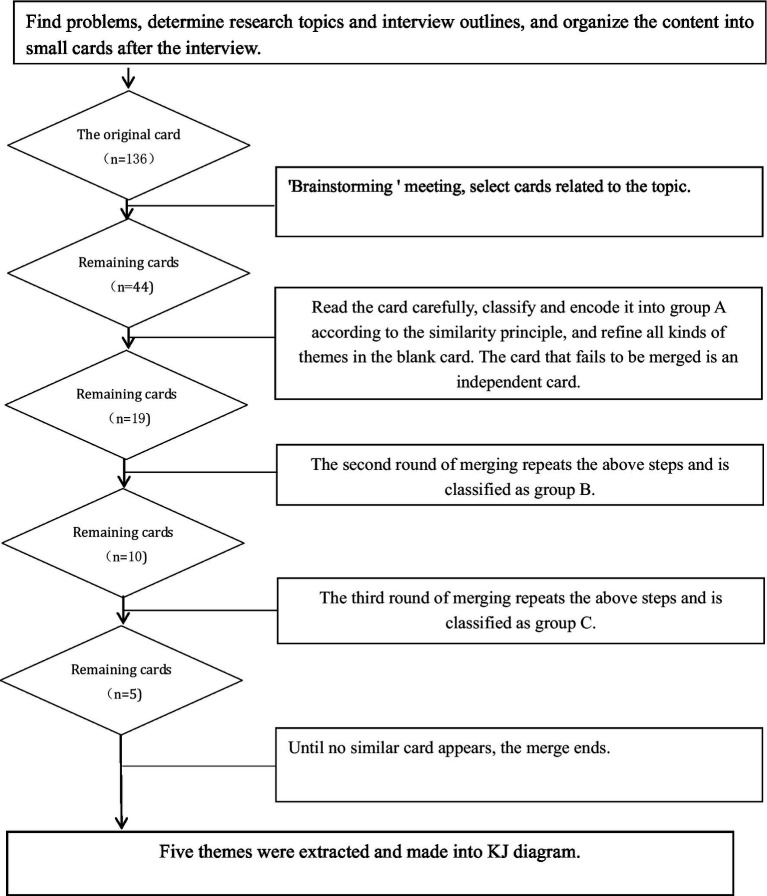
KJ method flow char.

Within 24 h following the interviews, the recordings were transcribed into text. All relevant materials were independently and thoroughly analyzed by two researchers, who listened to the recordings multiple times to ensure a profound understanding of the participants’ statements. Key phrases were selected from the interview data. These phrases were then coded into concise phrases or terms to extract the essential information. The participants’ original descriptions were encoded within brackets to preserve context. Upon completion of the entry extraction, the recorded materials were cross-compared by the two researchers. For this study, the KJ method was employed to analyze the data. The specific steps undertaken in this process are outlined below:

1. Experience: Two researchers independently and repeatedly reviewed the interview text data to grasp the core ideas intended by the interviewees.
2. Coding: Statements of significant importance were selected, condensed into concise items, and then summarized and encoded.
3. Card Filling: The coded statements were transcribed onto small cards (approximately 3 cm × 3 cm, cut from A4 paper).
4. Theme Refinement: All team members collectively reviewed the small cards three times, identifying those pertinent to the theme. Understanding the essence of each card, they grouped cards with similar content, labeling each group with a colored pen to represent a thematic focus. Cards with related content were further classified into intermediate group topics, marked on cards using a different color pen. Finally, closely aligned intermediate themes were amalgamated into overarching themes, resulting in the extraction of five major themes.
5. Construction of KJ Diagram: The five major themes were comprehensively analyzed and organized into a coherent KJ diagram. This diagram provided a global perspective on the themes extracted from the data. The KJ local example diagram (Figure 1) and the analysis flow chart (Figure 2) illustrate this methodological process in detail.

### 2.5 Quality control

Researchers have systematically studied qualitative research methods and acquired comprehensive expertise in the KJ research methodology. A professor, who underwent training in the KJ method in Japan, along with an enteral nutrition expert, actively participated in sorting, analyzing qualitative data, and provided valuable suggestions on extracting, classifying, and creating the KJ relationship diagram pertaining to the research topic. The graduate students within the group demonstrate proficiency in the qualitative research methodologies integral to the master’s program and excel in both communication and data organization. This study guarantees the thematic representativeness. During the selection phase, participants were chosen based on criteria including typicality, diversity, or homogeneity, thereby enhancing the authenticity of the dataset.

## 3 Results

### 3.1 General information

The study achieved thematic saturation after 11 interviews. The demographic data are as follows: 64% of participants were female, and 36% were male; the mean age was 38.27 ± 7.85 years; 27% of participants were from the neurology department, while 73% were from the neurosurgery department; 18% of participants held primary professional titles, 64% held intermediate professional titles, and 18% held advanced professional titles; 27% of participants were nursing supervisors, and 73% were nurses (Table 1).

**Table 1:** General characteristics of the participants (*n* = 11).

Numbering	Gender	Age(year)	Department	Professional title	Position
N1	Male	39	Neurosurgery	Intermediate	Nurse
N2	Male	41	Neurosurgery	Senior	Head Nurse
N3	Female	34	Neurosurgery	Intermediate	Nurse
N4	Female	36	Neurosurgery	Intermediate	Nurse
N5	Female	38	Neurology	Intermediate	Nurse
N6	Female	43	Neurosurgery	Intermediate	Nurse
N7	Male	32	Neurology	Junior	Nurse
N8	Male	28	Neurosurgery	Junior	Nurse
N9	Female	33	Neurosurgery	Intermediate	Nurse
N10	Female	58	Neurosurgery	Senior	Head Nurse
N11	Female	39	Neurology	Intermediate	Head Nurse

### 3.2 Comprehensive results

Initially, 136 original cards were generated, and a total of 44 cards relevant to the study’s theme were selected through a three-stage process of theme refinement. Subsequently, these cards were used to construct a KJ diagram, culminating in the identification of five overarching themes.

Five distinct themes emerged: (1) Preparation and evaluation before intubation; (2) Enhancement of intubation success rates; (3) Prevention of complications; (4) Management of complications and emergencies; (5) The acquisition of relevant knowledge and experience.

### 3.3 Theme 1: Preparation and evaluation before catheterization

For patients requiring gastrointestinal tube placement, the doctor should explain the procedure to the patients and their families, ensuring informed consent before catheterization. Nurses should assess the patient’s vital signs and check for any contraindications to intubation, selecting the appropriate gastrointestinal tube and insertion site based on the patient’s condition while ensuring the readiness of essential supplies.

N2: “*Typically, after the doctor explains the procedure, patients and families show high acceptance of the gastrointestinal tube.” N9: “Before intubation, it’s crucial to assess recent nasal surgeries or any trauma to the nose*.” N1: “*Patients generally need to fast for at least 4 h before intubation to avoid hindrances from food*.” N3: “*Gastrointestinal tube selection should consider the patient’s gastrointestinal function. Additionally, preparation of syringes and stethoscopes is necessary to assess tube positioning in the digestive tract*.”

### 3.4 Theme 2: Improve the success rate of catheterization

A diverse range of catheterization methods and operational skills significantly enhances the catheterization success rate while minimizing potential damage to the patient’s digestive tract. Nurses, based on the patient’s specific characteristics and their professional experience, select the appropriate location and technique for intubation. Various strategies and methods are employed to ascertain the correct positioning of the gastrointestinal tube during the catheterization process (20).

N8: “*During the gastrointestinal tube passage through the throat, encouraging the patient to perform swallowing movements—such as gently pressing the patient’s throat or administering 1-2 mL of water—facilitates intubation.*” N2: “*Based on my experience, palpating the patient’s abdominal wall and sensing vibration differences in various areas by injecting water and air into the gastrointestinal tube expedites head positioning of the tube*.” N1: “*If unable to extract intestinal fluid, a common practice involves injecting 20 mL of warm water into the gastrointestinal tube, followed by withdrawal and pH measurement*.”

### 3.5 Theme three: Standardize nursing operation to reduce the occurrence of complications

Nursing care for patients with indwelling gastrointestinal canal necessitates a dynamic structured assessment to tailor a patient-specific nursing plan (21). Employing standardized nursing procedures significantly enhances patient tolerance, a crucial factor in complication prevention and reduction of adverse events (22). N1: “*In neurology, we administer nutrient solutions through gastrointestinal canal while generally avoiding probiotics to prevent gastrointestinal symptoms*.” N2: “*For patients on long-term nutrient solution infusion via a nutrition pump, we consistently flush the tube with 20–30 mL of warm water every 4 h to prevent blockages*.” N10: “*When administering medication, it should be separated from the nutrient solution, with a flushing interval between the two*”.

### 3.6 Theme four: Scientific and timely treatment of complications

Implementing scientific and effective nursing interventions to address complications and emergencies minimizes patient harm and aids in their recovery. N2: “*In case of a blocked gastrointestinal tube, avoid forceful flushing to prevent tube rupture or intestinal perforation; gentle unclogging is essential. The success of recanalization largely depends on early detection*.” N6: “*The success of tube recanalization significantly relies on early detection; swift action increases the chances of successful intervention*.” N11: “*We employ single-day nasal tip and double-day nasal alar fixation to prevent pressure injuries*.” N1: “*Some patients may have allergic reactions to nasal paste; early observation for local skin reactions during tube fixation is crucial*.” N10: “*Diarrhea symptoms in patients tend to alleviate significantly upon discontinuation of nutrient solutions*.”

### 3.7 Theme five: Learning of relevant knowledge and accumulation of experience

Continual learning of pertinent gastrointestinal tube knowledge and daily intubation and catheter maintenance facilitate experience accumulation, enabling improved nursing care provision. N2*: “Our neurology department developed operational steps and videos for blind gastrointestinal tube insertion, revising them periodically.”* N3*: “Theoretical knowledge aligning with guidelines is valuable, but practical experience remains paramount in clinical work.”* N4*: “Some nurses pursued gastrointestinal tube learning due to their interest, frequently shadowing department experts to learn and consolidate experiences.”* N9*: “Qualified nurses for gastrointestinal intubation often convene to discuss, learn, and exchange intubation experiences”*. “N7: *“Occasionally, I refer to online resources for gastrointestinal tube nursing precautions, but department-led learning arrangements are more effective for me*.”

### 3.8 Analysis of the interaction between topics

Nurses’ ability to accurately assess patients’ physical conditions and the adequacy of equipment preparation before intubation determines the success of the intubation procedure and the effectiveness of managing complications in the future. The success of tube placement and whether mucosal damage occurs to the patient’s digestive tract during the intubation process impact the likelihood of future complications. The prevention and management of nasointestinal tube-related complications are interdependent, with standardized nursing procedures and rigorous handover systems able to prevent complications.

**Figure 3:**
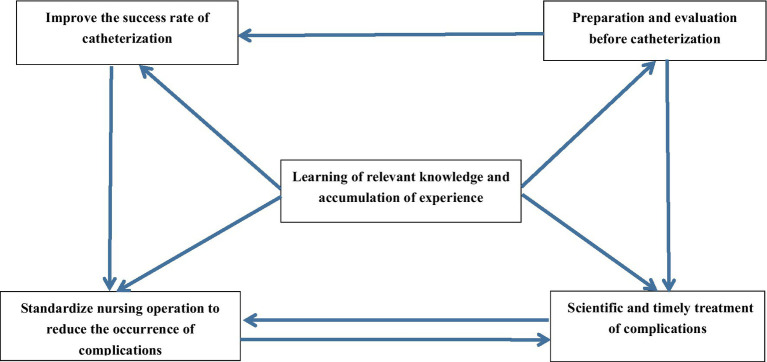
KJ correlation diagram.

Additionally, the scientific and effective management of complications can avoid further harm to patients from adverse events. Nurses’ learning of nasointestinal tube-related knowledge and accumulation of experience influence their ability to accurately assess patients’ physical conditions prior to intubation, improve intubation success rates through diversified techniques, and prevent and manage complications. Continuous learning of nasointestinal tube-related knowledge and daily maintenance of intubation and catheter care enable nurses to accumulate experience, thereby providing higher quality nursing care to patients. The KJ correlation diagram (Figure 3) is presented.

## 4 Discussion

### 4.1 Result analysis

In recent years, with the advancement and progress of the medical profession, nasoenteral tube feeding has gradually become one of the common methods for early enteral nutrition in critically ill patients in the field of neurology. Neurological patients often exhibit decreased neurological function, reduced memory, and poor capacity for understanding and compliance with treatment, thus making the care and management of nasoenteral tubes by nursing staff an increasingly important aspect affecting the recovery of neurological patients (23). During interviews, most nursing staff demonstrated a good understanding of gastrointestinal tube indications, contraindications, and the importance of tube selection, catheterization timing, and complication prevention and treatment. However, in clinical practice, the blind insertion technique lacks standardization and requires further refinement. Challenges persist for nurses in determining the head-end position and timing during catheterization (24). On one hand, the variability among individual patients and factors such as insufficient experience in catheter placement by nurses can lead to inconsistent catheter lengths. On the other hand, in clinical practice, due to time and financial constraints, it is often challenging to perform imaging studies simultaneously with tube insertion to assist with positioning, thereby prolonging the bedside blind insertion of nasoenteric tubes. Patients with indwelling gastrointestinal canal often encounter complications such as tube blockage and diarrhea (24), yet a standardized treatment method is lacking, warranting further research and improvement. Nursing staff should enhance their gastrointestinal tube-related knowledge and accumulate experience through daily intubation and care. This comprehensive approach aims to elevate pipeline nursing capabilities, ultimately delivering superior pipeline nursing care for patients.

#### 4.1.1 Establishing a scientific tube placement plan

Nursing research must be firmly grounded in clinical practice, prioritizing patient care. Research (25) has shown that nurses play a pivotal role in assessing the risks associated with gastrointestinal tube implantation and determining clear indications and contraindications before its implementation. Various types of nasojejunal tubes are chosen based on the intended purpose of their placement (24). Bloomer et al. (26) suggests that a scientifically devised catheterization plan should prioritize patient comfort while ensuring treatment efficacy, which is consistent with the results of this study.

Moreover, embracing a patient-centered nursing approach is essential. Chen et al. (26, 27) suggests that this encompasses humanistic care, health education, and effective nurse–patient communication. Nursing staff should comprehensively comprehend patients’ treatment expectations and their family dynamics. Conducting nutritional screenings to assess patients’ nutritional risks and promptly initiating enteral nutrition for high-risk individuals is crucial. McClave et al. (28) suggests that understanding the psychological state of both patients and their families is vital. In this study, explaining the feasibility and necessity of indwelling gastrointestinal canal to patients and families and devising tailored catheterization plans are imperative. In future clinical nursing endeavors, balancing the capabilities and requirements of both patients and caregivers is paramount for comprehensive and effective care.

#### 4.1.2 Implementing diverse tube placement methods and location assessment approaches

Studies (3, 29) indicate significant variations in the success rate of bedside blind insertion of gastrointestinal canal due to varying techniques and methods, primarily attributed to the absence of imaging equipment guidance. Enhancing catheterization success rates stands as a focal point in nursing staff research. This study reveals a consensus among neurology nurses regarding the crucial necessity of accurately determining the head-end position of gastrointestinal canal, influencing both catheterization success and safe tube use, aligning with prior research findings (30). Currently, multiple methods exist for determining the gastrointestinal tube’s head position in clinical practice, including abdominal X-ray positioning, digestive juice pH value determination, abdominal auscultation, and ultrasonic positioning (31). However, the efficacy levels of these detection methods vary. Studies (32, 33) has shown that the abdominal X-ray positioning method is considered the most accurate method, however, its inconvenience and associated patient transport and radiation risks limit its clinical utility. Hence, This study suggests that during catheterization, nurses should tailor their approach, considering the patient’s condition and medical setting, and employ diverse catheterization methods or techniques to assist in positioning, thereby augmenting catheterization success rates, which is consistent with previous research results (34).

#### 4.1.3 Scientifically and effectively preventing and managing complications

Research (35) has shown that the indwelling of gastrointestinal canal in neurological patients entails complexity and potential complications, so identifying tube-related complications, preventing their occurrence, and mitigating their impact on patients are persistent challenges for nursing managers. Bloomer et al. (26) suggests that a scientific management approach coupled with extensive clinical experience aids nurses in anticipating and recognizing pipeline-related complications promptly, enabling the formulation of effective countermeasures, which is consistent with the results of this study.

This study identified the risk of digestive tract injury and inadvertent insertion into the intracranial cavity during gastrointestinal tube placement for neurological patients. Early-stage complications post-catheterization commonly included diarrhea, while later-stage complications encompassed blockages and nasal pressure injuries. Hence, nurses should adopt gentle handling during catheterization to prevent mechanical harm and employ diverse fixation methods tailored to each patient’s condition (36).

Moreover, this study shows that strict control of infusion speed and solution temperature is crucial in the early stages to avert diarrhea caused by intolerance or disruptions in intestinal flora, this is consistent with some previous research results (37, 38). Consideration of prophylactic antibiotic use might be necessary. Researchers (39, 40) acknowledged pipeline, nutrient fluid, drug, and human factors as risks for tube blockage, consistent with this study’s findings. Avoiding drug infusion through the gastrointestinal tube is advisable. When essential, drugs should be fully dissolved before infusion (41). Regular pipe inspections before and after infusions, flushing to maintain openness, and standardized recanalization methods without forceful flushing are vital.

Improving departmental handover systems, implementing thorough ward rounds, promptly addressing complications, enhancing nursing quality, and facilitating patient rehabilitation are essential strategies to promote comprehensive patient care.

#### 4.1.4 Encouraging knowledge acquisition and experiential exchange

Neurological nurses generally believe that existing guidelines hold significant reference value when placing catheters and caring for patients with indwelling gastrointestinal canal. However, they often need to craft specific catheterization procedures and nursing strategies based on their own experiences and the unique clinical context. Unfortunately, many young nurses lack systematic training in gastrointestinal tube knowledge (42), resulting in prolonged catheterization times and an inability to promptly address complications due to insufficient clinical exposure.

Our study also highlighted a need for the improved initiative among nursing staff to acquire gastrointestinal tube-related knowledge. It’s crucial for nursing staff to proactively seek relevant information about gastrointestinal canal, such as the latest advancements in tube tip positioning methods and the prevention and treatment of gastrointestinal tube-related complications. To facilitate this, the department should regularly conduct introductory sessions on gastrointestinal tube intubation or nursing practices (43). These sessions will enable young nurses to enhance their knowledge, gather practical experience, and comprehensively enhance the nursing proficiency of neurological nurses caring for patients with indwelling gastrointestinal canal (44).

## 5 Limitations

The limitations of this study mainly come from the unavoidable subjectivity of qualitative research, which is closely related to the researchers’ cognitive ability, knowledge reserve and ability to analyze data. In addition, the unstructured nature of qualitative research makes it difficult to be used for statistical analysis or quantitative comparison. The number of tubes placed by nurses in each hospital may affect the results of the study. Confounding variables such as working years and work experience were difficult to manage. Young nurses were not included in the study because they did not have nursing experience in nasal jejunal tubes. Furthermore, this study only selected nurses from the departments of Neurology and Neurosurgery, potentially introducing bias in the analysis of qualitative data. Future research can expand the scope of sample inclusion, include more nurses in hospitals or institutions for analysis, gradually standardize the clinical operation of enteral nutrition, and ultimately achieve the effect of promoting patient rehabilitation.

## 6 Conclusion

The indwelling gastrointestinal tube, providing enteral nutrition for patients, stands as a crucial measure in enhancing the nutritional status of neurological patients (45, 46). This approach not only aids in improving prognosis and treatment success rates but also holds immense significance in restoring normal intestinal permeability, preventing complications like intestinal infections, bolstering immunity, rectifying metabolic disorders, and ultimately bettering the prognoses of neurological patients.

In this study, nurses from three hospitals were interviewed. While some findings aligned with prior research on aspects like preparation before catheterization, gastrointestinal tube positioning methods, and complication prevention, most interviewees expressed concerns about the effectiveness of gastrointestinal motility drugs before catheterization in improving success rates. There was also a lack of consensus on the best recanalization method for post-gastrointestinal tube blockage. In addition, the choice of auxiliary methods for bedside blind nasointestinal tube insertion remains to be further studied. Therefore, future research endeavors should aim to broaden the sample size, design high-quality randomized controlled trials for multi-center joint research, and further explore technologies to enhance the success rate of blind gastrointestinal intubation and the recanalization methods post-blockage.

## Data Availability

The original contributions presented in the study are included in the article/Supplementary material, further inquiries can be directed to the corresponding author/s.
