# Supplementary material for: Qualitative study on the ability of neurological nurses to manage patients with indwelling gastrointestinal canal
Source: Front Med (Lausanne). 2024 Dec 3;11:1403173. doi: 10.3389/fmed.2024.1403173 (PMC11653182; doi:10.3389/fmed.2024.1403173)
Supplement: Supplementary file 1 [file Table_1.DOCX]

**Manuscript:** Qualitative Study on the Ability of Neurological Nurses to Manage Patients with Indwelling Gastrointestinal Canal

**Consolidated criteria for reporting qualitative studies (COREQ): 32-item checklist**

Developed from:

Tong, A., Sainsbury, P., & Craig, J. (2007). Consolidated criteria for reporting qualitative research (COREQ): A 32-item checklist for interviews and focus groups. *International Journal for Quality in Health Care*, *19*(6), 349-357. <https://doi.org/10.1093/intqhc/mzm042>

| No. | Item | Guide questions/description | Comment | Reported on Page # |
| --- | --- | --- | --- | --- |
| 1. | Interviewer/ facilitator | Which author/s conducted the interview or focus group? | The first author conducted the interviews | 5 |
| 2. | Credentials | What were the researcher’s credentials? E.g. PhD, MD | Two nursing professors and five graduate students. | N/A |
| 3. | Occupation | What was their occupation at the time of the study? | The first author is a graduate student of nursing. Co-authored by two professors and four graduate students. | N/A |
| 4. | Gender | Was the researcher male or female? | Three male researchers and four female researchers. | N/A |
| 5. | Experience and training | What experience or training did the researcher have? | The research group consisted of seven members, including a professor who had studied the KJ method in Japan, an expert in enteral nutrition nursing research, and five nursing postgraduates. These graduate students have been systematically trained in the KJ method and have experience in qualitative research. | 3-4 |
| 6. | Relationship established | Was a relationship established prior to study commencement? | Researchers had no relationship with study participants prior to the study. | 4 |
| 7. | Participant knowledge of the interviewer | What did the participants know about the researcher? e.g. personal goals, reasons for doing the  research | Interviewer explained goals for doing the research. | 5 |
| 8. | Interviewer characteristics | What characteristics were reported about the interviewer/facilitator? e.g. Bias, assumptions,  reasons and interests in the research topic | Interviewer described his interest in the research. | N/A |
| 9. | Methodological orientation and Theory | What methodological orientation was stated to underpin the study? e.g. grounded theory,  discourse analysis, ethnography, phenomenology, content analysis | Using KJ method to analyze qualitative data. | 5 |
| 10. | Sampling | How were participants selected? e.g. purposive, convenience, consecutive, snowball | A total of 11 neurological nurses from 3 hospitals were selected for interviews in August 2023 by purposive sampling. The selection principle is : (1) Informed consent and willingness to participate in the study. (2) Proficient language expression and communication skills. (3) Experience with gastrointestinal tube catheterization or nursing. | 4 |
| 11. | Method of approach | How were participants approached? e.g. face-to-face, telephone, mail, email | Face-to-face | 5 |
| 12. | Sample size | How many participants were in the study? | 11 participants | 5 |
| 13. | Non-participation | How many people refused to participate or dropped out? Reasons? | No person refused to participate or dropped out | N/A |
| 14. | Setting of data collection | Where was the data collected? e.g. home, clinic, workplace | A separate closed room. | 5 |
| 15. | Presence of non-participants | Was anyone else present besides the participants and researchers? | No one else was present. | N/A |
| 16. | Description of sample | What are the important characteristics of the sample? e.g. demographic data, date | Participants included 4 men and 7 women, aged between 28 and 58 years, working in nursing for 3 to 39 years, with junior, intermediate and senior professional titles. | 4 |
| 17. | Interview guide | Were questions, prompts, guides provided by the authors? Was it pilot tested? | Semi-structured, dialogic interview guide that evolved over time was used | 5 |
| 18. | Repeat interviews | Were repeat interviews carried out? If yes, how many? | No repeat interviews were carried out. | 5 |
| 19. | Audio/visual recording | Did the research use audio or visual recording to collect the data? | All interview was audio recorded and transcribed. | 5 |
| 20. | Field notes | Were field notes made during and/or after the interview or focus group? | Field notes and reflective journals were completed after the interview | 5 |
| 21. | Duration | What was the duration of the interviews or focus group? | The interviews took between 30-40 minutes | 5 |
| 22. | Data saturation | Was data saturation discussed? | Yes, we discuss and focus on whether the new interview materials are meaningful to our research or promote the emergence of new research topics. | N/A |
| 23. | Transcripts returned | Were transcripts returned to participants for comment and/or correction? | No transcripts were returned to participants. | N/A |
| 24. | Number of data coders | How many data coders coded the data? | The first author and an enteral nutrition specialist coded the data and then reviewed and discussed it with collaborators | 5 |
| 25. | Description of the coding tree | Did authors provide a description of the coding tree? | The KJ method was used to encode, summarize and integrate the data. | 5 |
| 26. | Derivation of themes | Were themes identified in advance or derived from the data? | The theme is derived from qualitative interview data, and the KJ method is used to analyze the data. By selecting 44 cards related to the theme from 136 original cards, five themes are finally extracted through three stages of classification and coding.. | 5-6 |
| 27. | Software | What software, if applicable, was used to manage the data? | None | N/A |
| 28. | Participant checking | Did participants provide feedback on the findings? | Participants did not provide feedback. | N/A |
| 29. | Quotations presented | Were participant quotations presented to illustrate the themes / findings? Was each  quotation identified? e.g. participant number | Yes, quotes presented to illustrate themes and each quotation was identified while protecting participant confidentiality | 4-10 |
| 30. | Data and findings consistent | Was there consistency between the data presented and the findings? | Yes, consistency exists between data presented and the findings | 8-11 |
| 31. | Clarity of major themes | Were major themes clearly presented in the findings? | Yes, themes clearly presented | 8-11 |
| 32. | Clarity of minor themes | Is there a description of diverse cases or discussion of minor themes? | No minor themes discussed. | N/A |
